# Paraduodenal Hernia With Massive Intestinal Gangrene and Its Surgical Management: A Case Report

**DOI:** 10.7759/cureus.32008

**Published:** 2022-11-29

**Authors:** Kapil Rampal, Harkanwalpreet Kaur, Parampreet Sandhu, Harinder Singh, Ankush Bansal

**Affiliations:** 1 Surgery, Guru Gobind Singh Medical College and Hospital, Faridkot, IND; 2 General Surgery, Guru Gobind Singh Medical College and Hospital, Faridkot, IND

**Keywords:** jejunostomy, entrapped, short gut syndrome, gangrenous gut, paraduodenal hernia

## Abstract

Paraduonenal hernia constitutes more than 50% of internal hernia cases. It can result in perilous sequelae like gut ischemia and perforation. We report a case of a patient who presented with acute intestinal obstruction and peritonitis and was diagnosed as a case of complicated paraduodenal as an incidental finding on laparotomy.

A 26-year-old male patient presented with three days history of continuous severe incapacitating diffuse abdominal pain. The pain was associated with multiple episodes of bilious vomiting and absolute constipation. Patient had signs and symptoms of shock. Abdomen examination showed generalized peritonitis. Patient had deranged laboratory investigations. Abdominal X-ray showed acute intestinal obstruction. Patient was resuscitated and taken up for emergency laparotomy. Intraoperatively there was a long segment of gangrenous small bowel entrapped in the paraduodenal sac. Gangrenous gut was released from the sac and excised with proximal and distal ends fashioned as stoma through separate sites. Patient was managed with intravenous fluids with total parental nutrition. Patient gradually started on oral diet and jejunostomy output was refed through the distal stoma. Patient was discharged on postoperative day 14. Patient had uneventful early stoma closure at postoperative day 45 and now is on regular follow-up in the outdoor department.

Paraduodenal hernias are one of the rare causes of intestinal obstruction that is difficult to diagnose. Radiologic investigation like abdominal computed tomography (CT) scan can aid in diagnosis of paraduodenal hernia. Surgeons should have clear knowledge about abnormal anatomy of internal hernias and complications they can face during surgery.

## Introduction

Paraduodenal hernias are a subtype of internal hernias. An internal hernia is an abnormality in which an intra-abdominal organ protrudes through an opening in the peritoneum or mesentery. Paraduonenal hernia is uncommon and results from incomplete midgut rotation during the intra-uterine period. Paraduonenal hernia, however, is the most common type of internal hernia, constituting more than 50% of internal hernia cases [1,2]. It can manifest as intestinal obstruction or stay asymptomatic, and be found incidentally at autopsy or laparotomy. It can result in perilous sequelae like gut ischemia and perforation. Paraduodenal hernias are visible in radiological imagining [3-5]. Whenever surgeons come across extensive bowel ischemia, they always face a dilemma between the resection of all gangrenous bowel that may lead to short­gut syndrome, and leaving behind borderline viable intestine which may rupture and result in increased morbidity and possible mortality. We report a case of a patient who presented acute intestinal obstruction and peritonitis and was diagnosed with a case of complicated paraduodenal as incidental finding on laparotomy. We present a complicated case of paraduodenal hernia with extensive bowel gangrene resulting in short bowel syndrome in a young male patient.

## Case presentation

A 26-year-old male patient presented to the emergency department with three days history of continuous, severe, incapacitating and diffuse abdominal pain. Pain was associated with multiple episodes of bilious vomiting and absolute constipation. There was no previous history of postprandial fullness or bloating. 

The patient had signs and symptoms of shock (blood pressure 84/50mmHg, pulse rate 104 bpm). His abdomen was tense, diffusely distended and showed generalized peritonitis.

**Figure 1:**
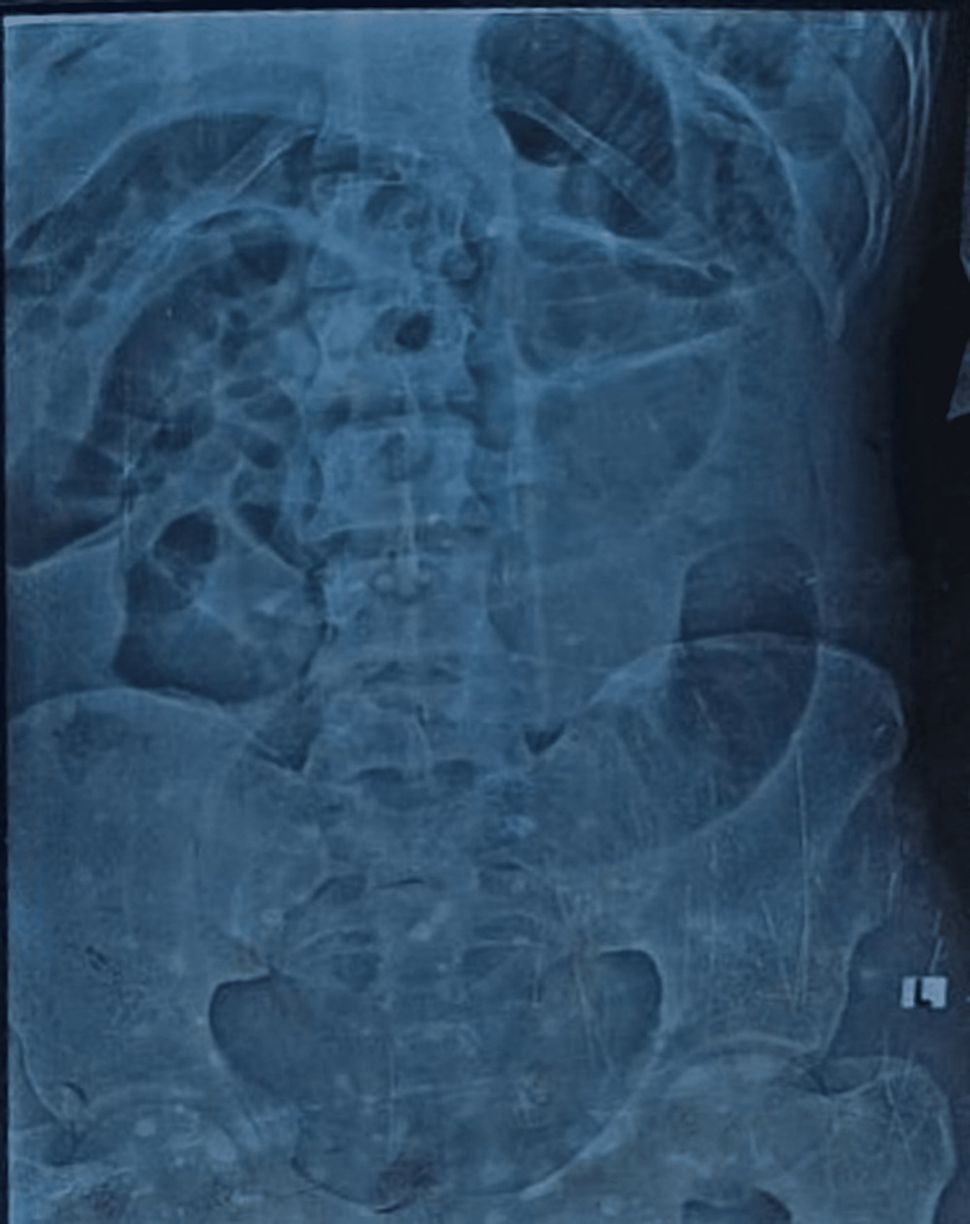
X-ray Abdomen (supine) showing dilated gut loops

**Figure 2:**
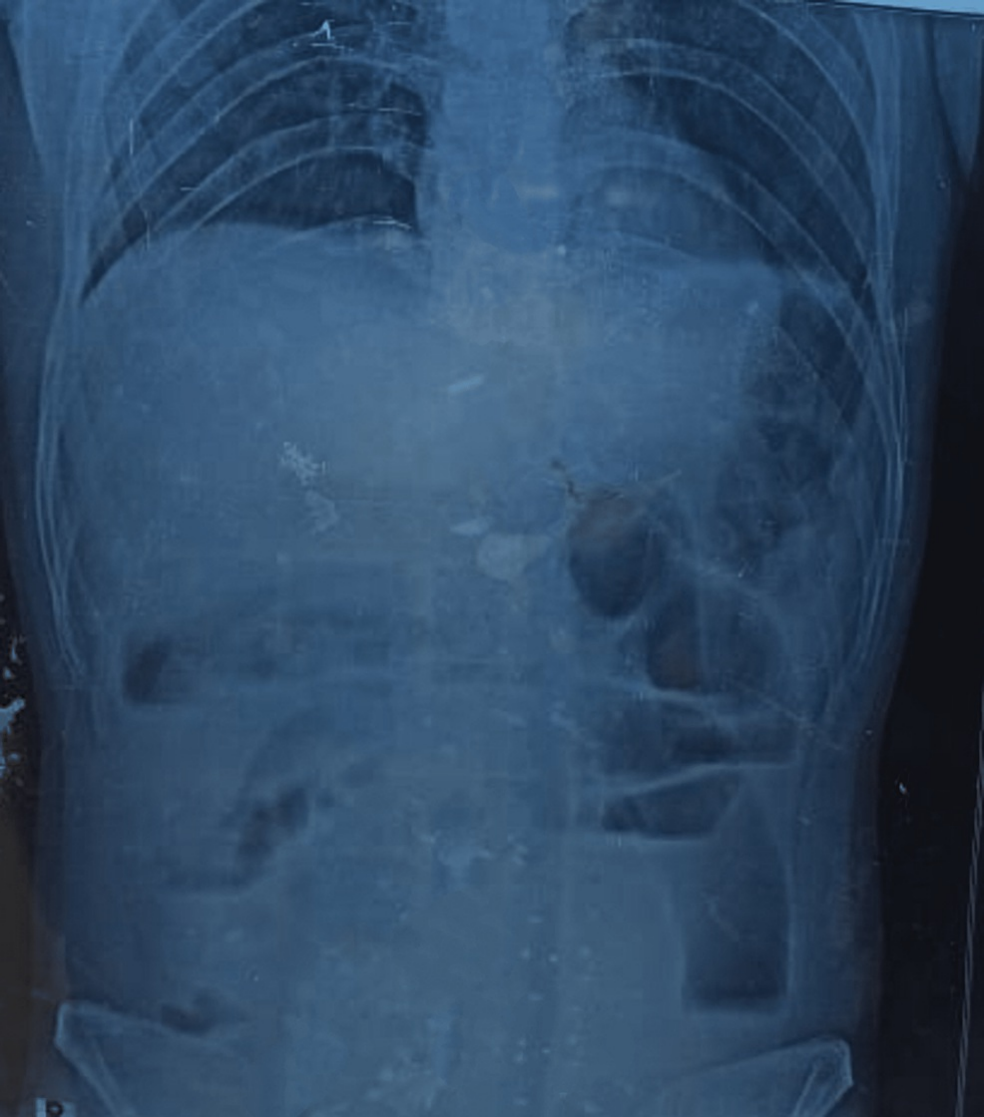
Erect X-ray Abdomen showing multiple air fluid levels

Laboratory investigations (Table 1) showed anemia, and deranged liver and kidney function tests. The X-ray (Figure 1) of the abdomen showed acute intestinal obstruction and no air under diaphragm (Figure 2). Intravenous contrast-enhanced computed tomography (CT) scan of the abdomen was not performed as the patient was in renal failure. The patient was resuscitated with intravenous crystalloids, two units of packed red blood cells and antibiotics. The patient was then taken up for an emergency laparotomy.

**Table 1:** Lab Investigations

Investigation	Value
Hemoglobin	5.65 mmol/l (Reference range-7.45-11.17 mmol/L)
White blood cells	2,7 × 10^9^/L (Reference range-3.5-12.0 X 10^9/L)
Platelet	124 × 10^9^/L (Reference range -150-400 X 10^9/L)
Urea	29.64 mmol/L (Reference range-2.5 -6.6 mmol/L)
Creatinine	203.37 umol/L (Reference range-60-120 umol/L)
Total bilirubin	35.91 umol/L (Reference Range-3-22umol/L)
Aspartate transaminase(SGOT)	84 IU/L (Reference range- 7-40 IU/L)
Alanine transaminase(SGPT)	40 IU/L (Reference range- 5-35-U/L)

**Figure 3:**
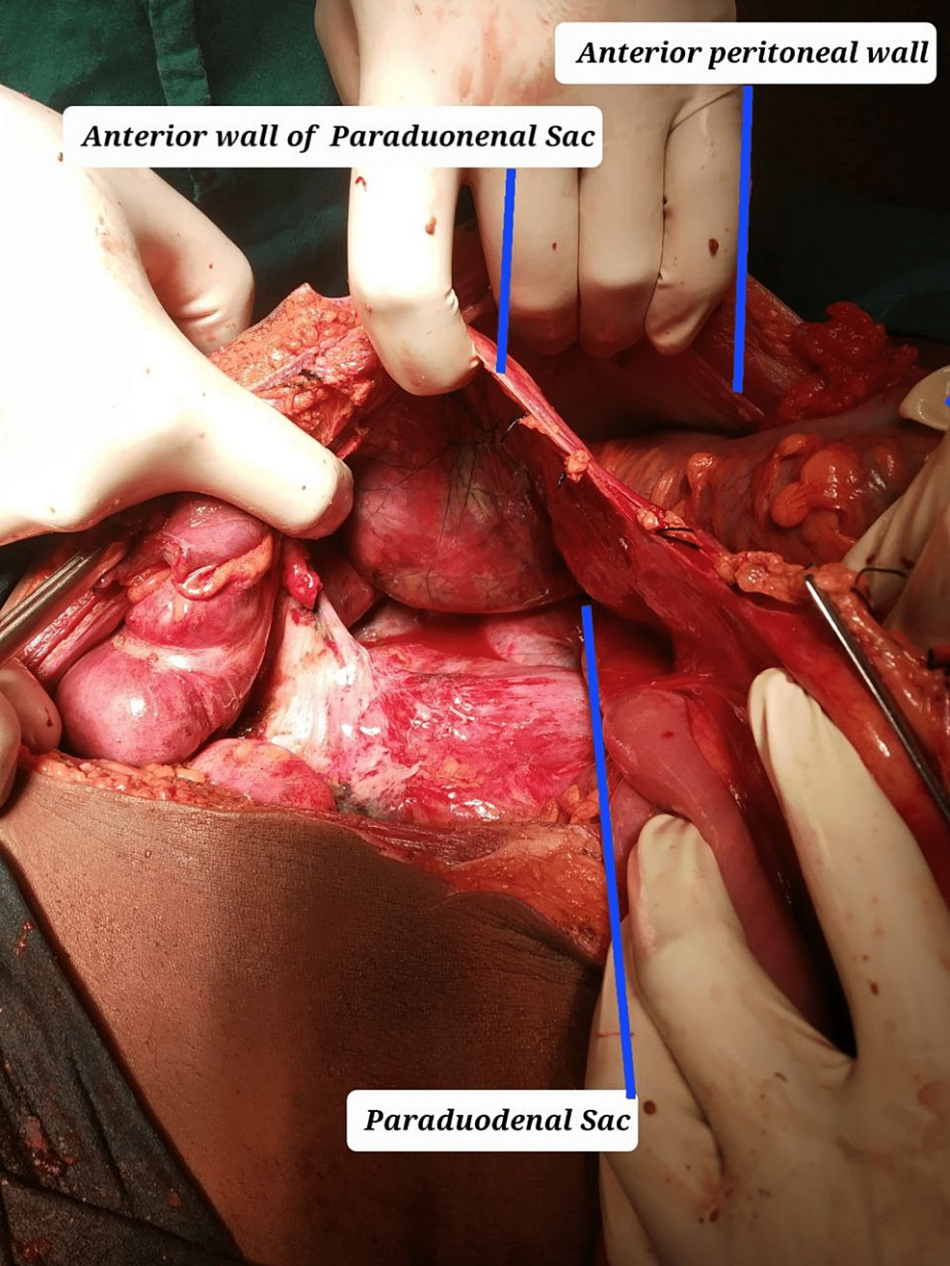
Paraduodenal sac

**Figure 4:**
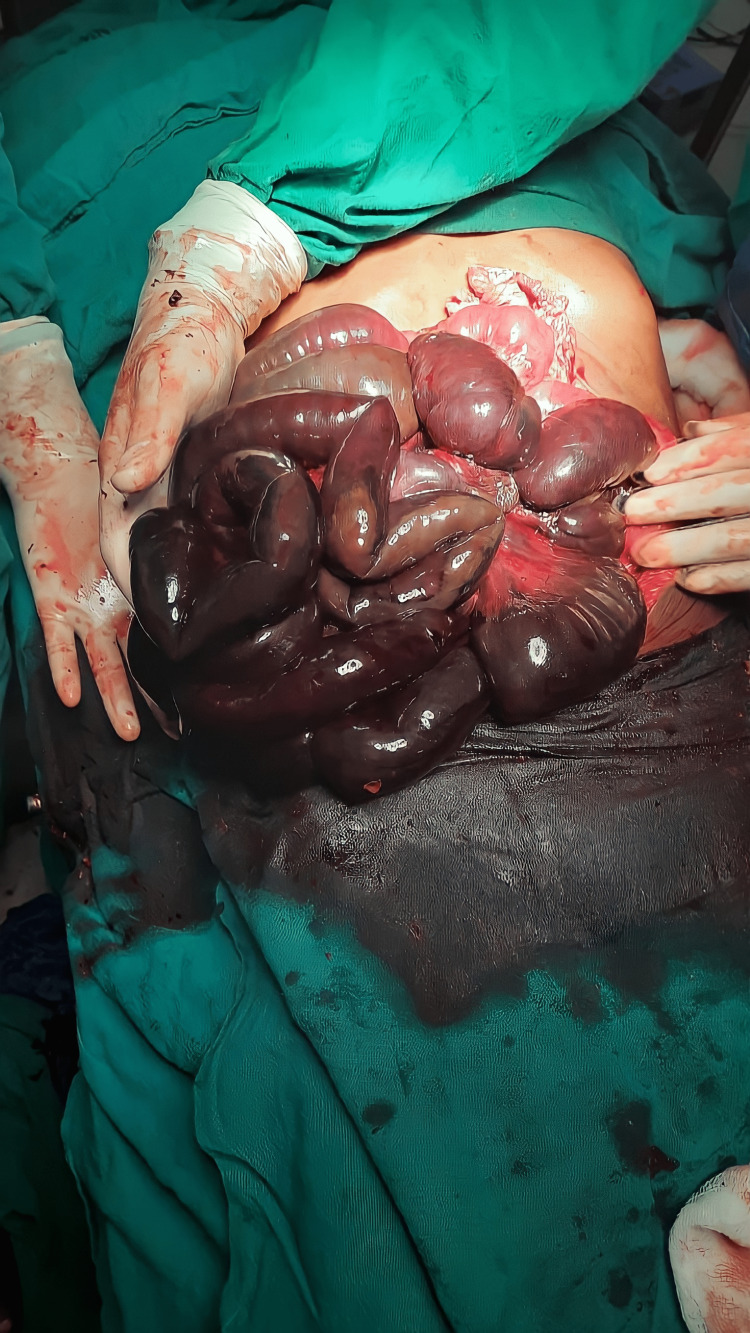
Gangrenous small bowel

**Figure 5:**
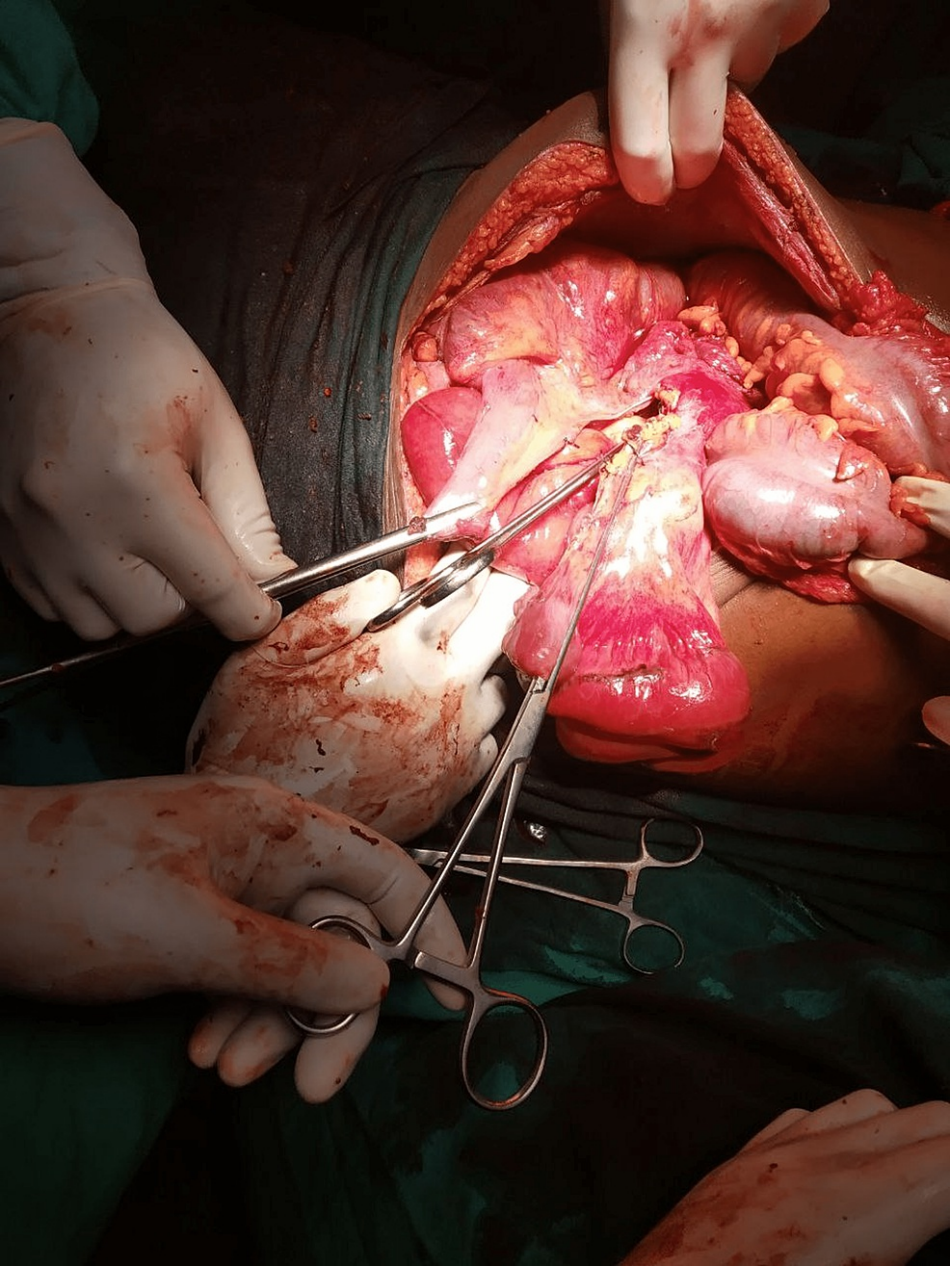
Proximal and distal end following resection of gangrenous small bowel

In intraoperative findings, the laparotomy was first met with an extremely foul smell emanating from the gangrenous small bowel. A part of the gangrenous bowel was seen entrapped in the paraduodenal sac (Figure 3), the anterior wall of which was formed by the mesentery containing blood vessels including the superior mesenteric artery. The duodenojejunal junction was located to the right of the vertebral column. The sac was excised sparing the mesenteric vessel and the entrapped bowel was released. The gangrene-involved segment of the small bowel 60 cm from duodenojejunal junction to 10 cm proximal to ileocaecal junction (Figure 4). The entire bowel length was inflamed and coated with pus flakes. The gangrenous bowel (5 metres) was excised and the proximal and distal ends (Figure 5) were fashioned as stoma through separate sites.

The patient was weighed on the second postoperative day for the baseline record. Short bowel syndrome occurred which was managed with refeeding, total parental nutrition (TPN) and early restoration of gastrointestinal continuity. For the initial five days the patient was managed with central venous pressure (CVP) and input-output-guided intravenous fluids with TPN. The patient gradually started on oral diet and the jejunostomy output was refed through the distal stoma. A dietician’s advice was taken. The jejunostomy output, which was 5-6 liters per day in the first two weeks, later settled to 2 liters per day. Loperamide (dose 4mg every eight hours) was used during this period. The patient was discharged on postoperative day 14 and followed up in the outdoor department. The patient had an uneventful early stoma closure on postoperative day 45 and now is on regular follow-up in the outpatient department.

## Discussion

Internal hernias are uncommon congenital abnormalities that can produce a varied range of complications related to intestinal obstruction, some potentially life-­threatening. These have 0.2%-0.9% incidence among all cases of bowel obstruction [6,7]. Approximately 53% of internal hernias are paraduodenal hernias (PDH) [8]. PDHs happen as a result of the defective reduction and rotation of the midgut during embryological development. Two theories have been propagated that explain the formation of congenital internal hernias. Moynihan’s theory suggests that ‘physiological adhesions’ occur when the bowel returns back to the abdomen and the common dorsal mesentery fuses with the posterior abdominal wall resulting in fusion folds and fossa formation. These fossae gradually increase in size, leading to PDH. Andrews’ theory also conceptualized Moynihan’s theory of fusion folds and fossae explained the congental defects of peritoneum, in which the small bowel is entrapped beneath the developing colon [6].

PDHs are a result of malrotation of the intestine and are more common in males than females (M:F = 3:1) [9]. On the basis of direction of the bowel herniation, PDH can be assigned as left and right paraduodenal hernia. Right paraduodenal hernias are rarer than left paraduodenal hernias (right:left = 1:3) [8]. Strangulation is more commonly seen in right paraduodenal hernias [10]. During embryological development, in right PDH, there is a counter-clockwise rotation of the midgut on the right side, entrapping the small bowel in a sac made by the peritoneum (the fossa of Waldeyer). With the inferior mesenteric vein lying posteriorly to the left and colonic mesentery, the caecum and ascending colon rotate the superior mesenteric artery lying anteriorly to the right [11]. About 20% of the cases of internal hernias require small intestinal resection due to bowel necrosis [12-14]. 

The diagnosis of paraduodenal hernia has been difficult whether patient is asymptomatic or symptomatic because symptoms are non-specific. It has been reported that 80% (8/10 cases) of the cases of symptomatic paraduodenal hernia were diagnosed preoperatively [12,13]. Abdominal contrast-enhanced computed tomography (CECT) has an important role in the diagnosis and management of internal hernias [2,15-17].

The important findings of paraduonenal hernia in abdominal CECT scan are small bowel loops clustering, well-circumscribed edge of hernia sac, and engorged mesenteric vessels. In the case of the left and right paraduodenal hernias, the CECT scan shows the inferior mesenteric vein (IMV) and superior mesenteric vein (SMV) direction of displacement. These are important clues to preoperative diagnosis [1,18].

We followed the operative methodology similar to the procedures mentioned in standard surgery textbooks. The open surgery was done. This included the mobilization of the right colon to the left by the Cattell Brasch maneuver, opening the hernia sac wide and placing the pre- and post-arterial segments of the intestine in the normal anatomical positions that they occupy after the first stage of rotation during embryonic development [19].

Resection of the strangulated bowel, if any, with primary anastomosis after surgically opening the hernial orifice has been reported by various authors [20]. This was not possible in our case as the patient had friable short length of bowel, and was in multiple organ failure. Hence, minimal and necessary in form of stoma formation was done to manage the patient.

## Conclusions

Paraduodenal hernias are one of the rare causes of intestinal obstruction, in addition to being difficult to diagnose. This should be suspected in patients in whom the cause of intestinal obstruction is not clear. Radiologic investigations like CT scans can aid in the diagnosis of paraduodenal hernia. It can be skipped if the patient is not stable and requires immediate surgical intervention. Surgeons should have a clear knowledge of the abnormal anatomy of internal hernias and the complications that they can face during surgery.
